# Oligodendrogenesis in the normal and pathological central nervous system

**DOI:** 10.3389/fnins.2014.00145

**Published:** 2014-06-12

**Authors:** Bilal El Waly, Magali Macchi, Myriam Cayre, Pascale Durbec

**Affiliations:** CNRS, Institut de Biologie du Développement de Marseille UMR 7288, Aix Marseille UniversitéMarseille, France

**Keywords:** oligodendrocyte, stem cells, myelin regeneration, multiple sclerosis, mouse models, adult brain plasticity

## Abstract

Oligodendrocytes (OLGs) are generated late in development and myelination is thus a tardive event in the brain developmental process. It is however maintained whole life long at lower rate, and myelin sheath is crucial for proper signal transmission and neuronal survival. Unfortunately, OLGs present a high susceptibility to oxidative stress, thus demyelination often takes place secondary to diverse brain lesions or pathologies. OLGs can also be the target of immune attacks, leading to primary demyelination lesions. Following oligodendrocytic death, spontaneous remyelination may occur to a certain extent. In this review, we will mainly focus on the adult brain and on the two main sources of progenitor cells that contribute to oligodendrogenesis: parenchymal oligodendrocyte precursor cells (OPCs) and subventricular zone (SVZ)-derived progenitors. We will shortly come back on the main steps of oligodendrogenesis in the postnatal and adult brain, and summarize the key factors involved in the determination of oligodendrocytic fate. We will then shed light on the main causes of demyelination in the adult brain and present the animal models that have been developed to get insight on the demyelination/remyelination process. Finally, we will synthetize the results of studies searching for factors able to modulate spontaneous myelin repair.

## Origin and fate of oligodendrocyte precursor cells

### Oligodendrogenesis from birth to adulthood

#### Generation of oligodendrocyte precursor cells during prenatal development and their fate in adult brain parenchyma

OLGs are the last cells to be generated after neurons and astrocytes during development. In the developing forebrain, several waves of OPC production and migration generate the entire OLG population. At E12.5 in mice, a first ventral wave of OPC production originates in the ganglionic eminence (Spassky et al., 2001; Tekki-Kessaris et al., 2001). As development progresses, a second and third wave of OPCs emanating from the lateral and caudal ganglionic eminences at E15.5 and from the cortex after birth, respectively, give rise to the majority of adult OLGs in mice (Kessaris et al., 2006). These OPCs spread to populate most of the future brain. Recent evidences have suggested that various waves of OPCs could myelinate distinct regions of the brain, suggesting the existence of distinct functional subpopulations of progenitors which could fulfill different functions (Tripathi et al., 2011). Interestingly in the brain as in the spinal cord, when any of these populations from different origins is ablated, neighboring populations quickly expand to generate the normal number of OLGs in the mature brain (Kessaris et al., 2006). These experiments suggest that OPCs compete during development and are functionally equivalent.

The OPCs generated during development in restricted areas have an amazing migration capacity and populate the brain and spinal cord to generate OLGs and myelinate the entire CNS during the postnatal life. A small fraction of OPCs generated during development are maintained as an immature slowly proliferative or quiescent state in the adult CNS (Dawson et al., 2003). These adult OPCs are present in all brain structures and represent 2–9% of the CNS cell population (Dawson et al., 2003). OPCs density does not perceptibly vary during adulthood but is higher in white matter than in gray matter (Dimou et al., 2008; Rivers et al., 2008). This difference is in part due to the higher rate of proliferation of OPCs in the white matter (Dimou et al., 2008). Notably gray and white matter OPCs also differ by their ability to differentiate into mature oligodendrocytes. While OPCs in white matter are proliferative and contribute to adult oligodendrogenesis, gray matter OPCs are quiescent or slowly proliferative and most remain in an immature state (Dimou et al., 2008).

Current evidence suggests that adult OPCs express the same markers as their developmental counterparts and appear morphologically similar (Franklin and Ffrench-Constant, 2008). Various markers are used to specifically identify adult OPCs, including the proteoglycan NG2 (Nishiyama et al., 1999) and the platelet derived growth factor receptor α (PDGFRα). They also express other markers such as the O4 antigen and the transcription factors Olig2, Olig1, and Nkx2.2 (Fancy et al., 2004). Since some of these markers can also be expressed by other cell populations (including synantocytes and endothelial cells of blood vessels) or alternatively maintained in later stages of OLG maturation, a combination of markers is often suitable to unambiguously identify OPCs in the adult CNS. But adult OPCs also differ from their developmental counterparts in some ways including growth factor responsiveness, migration capacities and longer cell cycle time (Wolswijk and Noble, 1989; Wren et al., 1992; Psachoulia et al., 2009). In the adult brain, half of the OPCs are cycling in physiological condition (average period of around 1 week) while the remaining OPCs are quiescent or divide more slowly (Rivers et al., 2008). This suggests that adult OPCs could represent a heterogeneous population with possible distinct functions. Various studies have demonstrated that gray and white matter OPCs have differing resting membrane potentials and ion channel expression profiles (Yuan et al., 2002; Karadottir et al., 2008). Two classes of OPCs have been identified in the adult brain: one lacking voltage-gated Na+ channels completely, whereas the other expresses voltage-gated Na+ current and generates trains of action potentials. OPCs that generate action potentials are capable of sensing the neuronal activity in the environment by receiving synaptic inputs from axons and are more sensitive to ischemia (Karadottir et al., 2008).

Other studies have suggested that OPCs properties could be in part due to environmental cues. For example many reports have illustrated differences in proliferation and differentiation capacity of OPCs depending on brain areas. A recent study has shown that OPCs from white matter can efficiently differentiate into myelinating oligodendrocytes independent of the area they were transplanted in while OPCs purified from gray matter do so less efficiently (Vigano et al., 2013). The gray matter has been shown to decrease OPC proliferation and to arrest OLG differentiation while white matter environment maintains division and favors OLG maturation (Dawson et al., 2000; Dimou et al., 2008; Rivers et al., 2008).

#### Oligodendrogenesis in postnatal subventricular zone

After birth and in the adult brain, OPCs are continually produced by adult NSCs located in the SVZ (Menn et al., 2006). SVZ stem cells generate fast dividing transient amplifying cells (C cells) which in turn generate neuronal progenitors migrating toward the olfactory bulb (OB) and differentiating as interneurons (Lois and Alvarez-Buylla, 1993; Doetsch et al., 1999). Neuronal progenitors migrate tangentially along the rostral migratory steam (RMS), a well-defined pathway from the SVZ toward the OB (Kirschenbaum et al., 1999). A small fraction of C cells generate OPCs which migrate radially out of the SVZ into the overlying white matter and cortex, (Suzuki and Goldman, 2003; Menn et al., 2006) for review see (Cayre et al., 2009). A recent analysis using live imaging and single-cell tracking technologies in the SVZ has shown that a single NSC exclusively generates OLGs or neurons, but never both (Ortega et al., 2013). The number of OLGs formed is consistently lower compared with the number of neurons that migrate and differentiate in the OB (Menn et al., 2006). The ratio of OLG/neuron production is dependent on the dorsoventral and rostrocaudal levels in the SVZ. In the adult brain where this aspect has been extensively studied, more OLGs are produced from the dorsal part of the SVZ (facing the corpus callosum) than from the ventrolateral part of the structure (facing the striatum) (Ortega et al., 2013). Furthermore, along the rostrocaudal axis of the brain, a higher number of OLGs are produced in the posterior part of the SVZ (1 OLG/3 neurons) compared to rostral SVZ (1 OLG/30 neurons) (Menn et al., 2006). This regionalization of the SVZ for OLG production is under the influence of environmental factors including Wnt dorsally (Ortega et al., 2013) and BMP ventrally (Colak et al., 2008) respectively favoring and inhibiting OPC specification in the adult brain. The OLG/neuron ratio also changes depending on the age of the animal: while the number of OPCs produced in the SVZ after birth is important leading to numerous and active production of OLGs in the corpus callosum and cortex, this number is drastically reduced in the adult brain. Then, in the aging brain, this low level of oligodendrogenesis is maintained while neuron production in the OB is compromised and RMS cytoarchitecture altered (Capilla-Gonzalez et al., 2013).

#### Oligodendrocyte determination

***Extrinsic factors***. The relative intensity of Shh, BMP and Wnt/β-catenin signals during development is a critical determinant of oligodendrogenesis. In the spinal cord OPC commitment and production is tightly regulated by these patterning molecules inducing the expression of specific sets of transcription factors along the dorso-ventral axis. The pMN domain marked by the Olig2 expression first generates motor neurons and then produce most of the OPCs. This switch is tightly dependent on the dose of Shh (Dessaud et al., 2010) regulated by heparan sulfate dependent gradient formation (Touahri et al., 2012) and by the Notch/delta pathway limiting the number of motoneurons produced and allowing OLG commitment (Kim et al., 2008). The dorsalizing factors, BMPs and Wnt also regulate this decision by repressing the OPC fate (See and Grinspan, 2009). The neuroglial switch in the pMN induces the expression of specific genes involved in OPC determination. Although Olig2 is expressed in motoneuron precursors in the pMN, its expression is down regulated in differentiating neurons and then this factor becomes specific to oligodendrocytic lineage.

The same molecular cues act in the brain, where the presence of Shh induces the formation of ventrally derived OPCs in the forebrain (Tekki-Kessaris et al., 2001). The dorsally expressed Wnt/β-catenin and BMP pathways antagonize the effect of Shh. In opposite to the inhibitory effect of Wnt in the spinal cord (Shimizu et al., 2005), Wnt3 a factor belonging to the same family plays a crucial role in promoting OLG specification in the adult SVZ (Ortega et al., 2013).

In the adult brain, the germinal niche (i.e., SVZ) possesses specialized properties regulating stem cell proliferation and commitment toward specific lineages (Ihrie and Alvarez-Buylla, 2011). In particular, numerous extrinsic cues secreted by blood vessels or associated to the extracellular matrix have been described in the niche to favor OLG formation in physiological conditions. For example, endothelial cells of the blood vessels have the capacity to promote OPC commitment (Chintawar et al., 2009; Plane et al., 2010). This effect is mediated in culture by the production of specific chemokines including MCP1 (Chintawar et al., 2009). Among components of the extracellular matrix that could regulate OLG production, laminin plays a crucial role (Relucio et al., 2012). Mice lacking laminin α2-subunit show a reduction in the number of OPCs produced in the SVZ and present in the adjacent corpus callosum (Relucio et al., 2012). The presence in the area of heparan sulfate (Liu and Casaccia, 2010; Mercier and Arikawa-Hirasawa, 2012) could also suggest an important role of proteoglycans in the regulation of morphogene and/or trophic factor activity (Hacker et al., 2005). Most trophic factors present in the SVZ, such as PDGF (Jackson et al., 2006) and EGF (Aguirre et al., 2007), regulate OPC proliferation and/or maturation but do not directly act on OLG determination in this area.

***Transcription factors and epigenetic***. Besides these environmental cues, which are important to define the domains that will generate OLGs in CNS, cell commitment toward the OLG lineage is regulated by the dynamic interaction between transcription factors and epigenetic factors including microRNA (miRNA) (Liu and Casaccia, 2010).

*Olig2*. The best-defined transcription factor regulating OLG production is the basic helix-loop-helix transcription factor Olig2 induced by ventrally secreted Shh in the spinal cord (Lu et al., 2002). Olig2 is expressed throughout the oligodendrocyte lineage from OPCs to myelinating oligodendrocytes. Olig2 inactivation during development leads to a reduced production of OPCs in most CNS regions (Lu et al., 2002; Zhou and Anderson, 2002; Ligon et al., 2006) and induction of Olig2 expression in neuroepithelium leads to an increased generation of OPCs in the CNS (Maire et al., 2010). Interesting data from Olig2-null mice show that some OPCs are still generated in the hindbrain, most likely through compensation by Olig1 (Lu et al., 2002). Olig2 is thus necessary and in some brain region sufficient for OPC determination. After birth, Olig2 is restricted to few cells in the SVZ representing a subpopulation of C cells which will be committed to the OLG lineage and migrate to the corpus callosum and cortex (Menn et al., 2006). In the SVZ, Olig2 promotes oligodendrogenesis by opposing Pax6 function that favors neuronal progenitors generation (Hack et al., 2005). In addition, the interactions between Olig2 and inhibitor of differentiation (ID) family of helix-loop-helix transcriptional inhibitors mediate the inhibitory effects of BMP4 on oligodendrogenesis (Samanta and Kessler, 2004).

*Ascl1/Mash1*. The bHLH factor Ascl1/Mash1 plays an important role in OLG specification. Ascl1 knockout mice show decreased OPC generation in both the brain and spinal cord at early stages (Parras et al., 2007; Sugimori et al., 2008). Nevertheless, relatively normal numbers of OPCs are ultimately formed in these mice may be due to the redundant function of Ascl2 and 3 also expressed in oligodendrocyte lineage in the developing CNS (Sugimori et al., 2008). After birth, Ascl1 expression is restricted to neural progenitors (C cells) and OPCs in the SVZ (Nakatani et al., 2013). In this context, ablation of Ascl1 gene results in a strong reduction of OLG production and leading to astrocyte overproduction by SVZ progenitors (Nakatani et al., 2013). In the adult brain, Ascl1 is required in oligodendrogenesis both under normal physiological conditions and after demyelinating lesions. A recent study reported that while Ascl1/Mash1 and Olig2 expression oscillate in neural progenitors, their stable expression is observed during determination in neuronal and oligodendrocyte progenitors respectively (Imayoshi et al., 2013).

*Nkx and Sox families*. Other transcription factors belonging to the Homeobox and Sox family are also participating to oligodendrogenesis, but loss of function analyses have shown a non-essential or redundant role of these factors for OPC specification (Richardson et al., 2006). For example, motoneurons and OLGs generated from the pMN domain are eliminated in the Nkx6.1/Nkx6.2 double mutants demonstrating an essential role for these factors during development (Cai et al., 2005; Vallstedt et al., 2005). Among the sox gene family, Sox9, Sox8, and Sox10 are all expressed during OLG development. Mice lacking Sox9 have a reduced number of OLGs and astrocytes indicating a requirement for this factor in general glial specification (Stolt et al., 2003). Sox8/ Sox9 double inactivation leads to a complete absence of OLGs suggesting a redundancy between the two factors (Stolt et al., 2003). Despite the fact that Sox10 is often used as a specific marker for oligodendrocyte lineage cells, the loss of this gene has no effect during determination but results in failure in terminal cell differentiation (Stolt et al., 2004).

In summary, despite expression of numerous specific transcription factors early during OPC determination most of them such as Nkx2.2, Sox10, and Olig1 are not required for lineage specification. Among all, Olig2 seems to be the only one absolutely required for the specification of the lineage. In this context, Olig2 is the only transcription factor absolutely required for direct mouse fibroblast reprogramming (Najm et al., 2013; Yang et al., 2013).

*miRNA*. miRNAs have been shown to play important roles in both brain development and the regulation of adult neural cell functions. Over the last few years, some specific miRNAs were found to participate in the regulation of OLG differentiation and myelin maintenance, as well as in the pathogenesis of demyelination diseases (for review see Liu and Casaccia, 2010; Li and Yao, 2012). Various studies have identified sets of miRNAs expressed specifically by OLGs (Lau et al., 2008; Jovicic et al., 2013). Two independent laboratories used targeted deletion of Dicer1 in OLGs to disturb microRNA processing. They showed that preventing miRNA processing disrupts normal brain myelination despite an expansion of the OPC pool (Dugas et al., 2010; Zhao et al., 2010). By contrast, miR-7a, one of the highly enriched miRNAs in OPCs, promotes the generation of OPCs when overexpressed in neural progenitors *in vitro* and *in vivo* during brain development. Blocking the function of miR-7a in differentiating neural progenitors led to a reduction of OPCs and an expansion of neuronal progenitors (Zhao et al., 2012).

*Histone modifications*. Beside miRNA, other epigenetic mechanisms including histone modifications can regulate OPC production from NSCs (Liu and Casaccia, 2010). The activity of histone deacetylases (Hdac) is for example important for OLG determination in the neonate SVZ. The ability of a stem cell to choose alternative fate and to differentiate as OLG progenitors depends on the activity of Hdac *in vitro* (Siebzehnrubl et al., 2007; Foti et al., 2013). The involvement of histone methylation in the transition from stem cell to OPC is also suggested by evidence on the role of Enhancer of zeste homolog 2 (Ezh2), a polycomb group protein involved in gene silencing via histone methylation. Overexpression of Ezh2 increases the number of OLGs at the expense of astrocytes, whereas its silencing produces opposite effects (Sher et al., 2008).

Epigenetic regulation of OLG determination and maturation is still a field to investigate and will provide in the future a better understanding of oligodendrogenesis. It could offer new targets for myelin repair (Liu and Casaccia, 2010).

### Oligodendrocyte precursor cells and myelin plasticity in adulthood

#### Oligodendrocyte precursor cells as multipotent stem cells

It has been suggested that adult OPCs may represent multipotent stem cells in the adult brain. Purified OPCs in specific culture conditions can be reverted to an immature NSC state: cells can self-renew and give rise to neurons, astrocytes, and OLGs (Kondo and Raff, 2000; Belachew et al., 2003). Nevertheless, *in vivo* analysis showed that OPCs in the hippocampus generated predominantly OLGs, rarely neurons but never astrocytes (Belachew et al., 2003). OPCs isolated from newborn mouse brain may generate hippocampal neurons when transplanted into the SVZ (Aguirre et al., 2004). These studies suggested that OPCs generated during the early postnatal life are multipotent *in vitro* and can generate, beside OLGs, a small number of neurons *in vivo*. Cre-Lox cell fate-mapping studies in mouse have recently entered the debate. Genetic fate tracing using mice in which tamoxifen-inducible Cre is expressed in OPCs has shown that while OPCs generate both astrocytes and OLGs during development, in the postnatal brain the capacity of these cells to generate astrocytes is lost and adult OPCs produce exclusively OLGs (Dimou et al., 2008; Rivers et al., 2008; Kang et al., 2010; Zhu et al., 2011). Altogether these analyses confirm the idea that after birth OPCs do not generate astrocytes. Rivers et al. (2008) reported the generation of a small subpopulation of neurons in the cortex but this observation was due to pitfalls of the genetic fate mapping using inducible Cre-recombinaison (Richardson et al., 2011; Clarke et al., 2012). Although the multipotent capacity of OPCs is still a matter of debate (Richardson et al., 2011), it seems most likely that adult OPCs remain quiescent or slowly proliferative progenitors and differentiate exclusively as myelinating OLGs in the adult brain.

#### White matter plasticity in adulthood

One of the most striking advance in this field has been the demonstration that myelination perpetuates in the adult brain. The use of inducible Cre-Lox fate mapping has allowed researchers to genetically label OPCs in the adult brain and to follow their progeny for a prolonged period of time and demonstrate their capacity to myelinate continuously throughout normal adult life (Dimou et al., 2008; Rivers et al., 2008). It is demonstrated that 20% of all OLGs in the adult mouse corpus callosum were generated after 7 weeks of age and that mature myelinating OLGs were still generated until at least 8 months of age (Rivers et al., 2008). A complementary analysis has shown that OLGs are still generated at a low rate after 8 month of age in mouse brain (Psachoulia et al., 2009). Very little is known about the factors controlling adult oligodendrogenesis. Physiological stimulus such as volontary physical exercice can promote cell cycle exit and terminal differentiation of adult OPCs (Simon et al., 2011). Interestingly, a recent study demonstrated the impact of neuronal activity on myelination in the adult brain (Gibson et al., 2014).

In humans magnetic resonance imaging showed that white matter volume increases up to the age of 19 years (Giedd and Rapoport, 2010) and that exercising complex skills such as piano playing can further increase myelination (Bengtsson et al., 2005).

Apart from the case of myelin repair which will be further described below, one important issue now is to understand what is the function of this constant production of myelinating OLGs over life in the brain. Myelination of fiber tracts is not homogeneous in all structures (Sturrock, 1980; Dangata and Kaufman, 1997). For example, the corpus callosum is known to contain a mixture of myelinated and unmyelinated axons in rodent (Sturrock, 1980) while the optic nerve is predominantly composed of myelinated axons (Dangata and Kaufman, 1997). Thus, newly form OLGs could be involved in the myelination of naked axons in such structure or alternatively, new OLGs could participate to a physiological turnover of myelin compartment to replace dying OLGs in the entire brain. A recent study (Young et al., 2013) has tackled this issue trying to distinguish between these two hypotheses. Crelox fate mapping has demonstrated that the number of newly formed OLGs is independent of the level of myelination of the structure. OLG accumulation is faster in the corpus callosum (contening a mixture of myelinated and naked axons) than in the optic nerve (almost exclusively formed of myelinated axons) but the overall production of long-term-surviving OLGs over a 10 months period is similar in the highly myelinated optic nerve and the partially myelinated corpus callosum (Young et al., 2013). Thus, myelin replacement is independent of the degree of axon myelination in the structure. Beside, the authors observed that adult-born OLGs produce more and shorter internodes (Young et al., 2013). Overall they concluded that most of the new OLGs produced in the adult optic nerve are engaged in myelin remodeling, either by replacing dying OLGs or by adding additional myelin internodes in such a way that the total number of myelinating cells increases without a concomitant increase in the total length of myelin sheath (Young et al., 2013). Another interesting aspect of this analysis is the demonstration that myelin in the adult brain is thinner (larger g-ratio) than myelin synthesized in neonate brain (Young et al., 2013). This analysis could suggest that thinner myelin and shorter internodes associated with remyelination (see below) might be a general property of adult myelination rather than a feature of the regenerative process.

One important step in the future will be to elucidate the functional consequences of myelin plasticity in the adult brain.

## Oligodendrogenesis in the diseased brain

### Disorders leading to demyelination

Apart from well-characterized primary demyelinating diseases such as multiple sclerosis, demyelination also occurs in a large variety of CNS insults and pathologies. This is in part due to high OLG susceptibility to oxidative stress, excitotoxic damage and inflammatory cytokines, which often go along with trauma, lesions or infections.

#### Basis of oligodendrocyte susceptibility

The intrinsic function of OLGs contributes to their high susceptibility to injuries. Indeed, OLGs show the highest metabolic rates among all brain cells in order to produce but also to maintain very high volume of membranes which can represent up to 100 times the weight of the cell (Morell and Toews, 1984). Cell respiration in OLGs has been reported to be twice higher than in neurons (Cammer, 1984). Furthermore, OLGs are paradoxically poor in gluthatione (Thorburne and Juurlink, 1996) which is a major anti-oxydant. Thus, OLGs are predisposed to cell death vulnerability in conditions of metabolic stress (Connor and Menzies, 1996).

OLGs are also exposed to excitotoxicity since their glutamate receptors are more permeable to calcium (Benarroch, 2009). Glutamate plays major roles in white matter disorders, for instance its concentration is increased in plaques and normal appearing white matter of MS patients (Werner et al., 2001; Srinivasan et al., 2005). In conditions of energy breakdown, reversal of the excitatory amino acid transporters (EAAT) may lead to glutamate release, contributing to glutamate-induced cytotoxicity via calcium-triggered NO and O2- synthesis, peroxynitrite production and finally OLG apoptosis (Benarroch, 2009).

In pathological conditions, activated microglia also contribute to increase excitotoxicity by secreting glutamate, peroxynitrite and TNFα (which in turn inhibits EAAT activity and activates apoptosis) (Li et al., 2005; McTigue and Tripathi, 2008).

Such susceptibility of OLGs partly explains that many pathological situations can affect OLGs and lead to demyelination. In the following section, we will distinguish demyelination occuring secondary to a pathology that does not primarily target OLGs and diseases that are considered as true primary demyelinating diseases.

#### Demyelination secondary to a primary non-demyelinating pathology

***Trauma and ischemia***. Primary mechanical damage (strain forces on tissues, compressions, distortions) is immediately followed by micro hemorrhages and focal impairment of the cerebral blood flow that trigger secondary inflammatory and degenerative processes involving NO, glutamate and free radicals production (Carlson et al., 1997; Golding, 2002). Posttraumatic OLG death has been reported both in animal models (Flygt et al., 2013) and in postmortem human brains (Shaw et al., 2001). It is however not always clear whether this is a primary event or if it is secondary to axonal degeneration. Dynamic of myelin loss and neuronal death suggest that this could vary depending on the type of trauma. Similarly, ischemia leads to hypoxia-induced cell death in the brain. Ischemia occurring in subcortical white matter account for up to 25% of all stroke subtypes (Bamford et al., 1991; Schneider et al., 2004) and the incidence of such strokes increases with age (Vernooij et al., 2007). According to the mechanisms of OLG susceptibility described above, it could be predicted that OLGs would be highly vulnerable to ischemia. Indeed, OLG swelling and vacuolization appear as soon as 3 h after middle cerebral arteria occlusion, followed by processes retraction and cell death within 24 h (Pantoni et al., 2006; McIver et al., 2010). Different studies suggest that improving remyelination after trauma or ischemia could be an interesting strategy for improving functional recovery (Plemel et al., 2014).

***Neurodegenerative diseases***. Recently, demyelination has also been associated to a number of neurodegenerative diseases such as Alzheimer and Huntington, and OLG alterations are observed across most psychiatric disorders including autism, schizophrenia and depression (for review see Edgar and Sibille, 2012). Analyses from postmortem brain tissue of Alzheimer patients showed a decrease in the protein levels of MBP, PLP and CNPase (Vlkolinsky et al., 2001; Roher et al., 2002), together with significant regional atrophy of the corpus callosum (Hampel et al., 1998). More recent studies suggest that white matter abnormalities represent an early feature of Alzheimer (far before neurofibrillary tangles and clinical symptoms) and might also contribute to the pathogenesis of the disease (Sachdev et al., 2013). Indeed, Aß peptide accumulation probably contributes to white matter defects since a direct cytotoxic effect on OLGs has been demonstrated *in vitro*, with DNA damage, mitochondrial dysfunction and loss of cytoskeleton (Xu et al., 2001; Lee et al., 2004). Furthermore, a strong correlation between Aß levels and myelin damage was found in patient's brains postmortem (Roher et al., 2002). Detection of white matter abnormalities could thus help to early diagnosis of Alzheimer's disease and even be a target for the development of new therapeutic strategies.

A recent study showing a neuron-to-OLG transfer of abnormal proteins may point to possible mechanisms leading to OLGs death in neurodegenerative diseases (Reyes et al., 2014).

***Irradiation***. Brain irradiation can be useful to treat head and neck tumors. However, repeated or high doses of radiation may also have deleterious effects on the central nervous system such as vascular changes and white matter pathology ranging from demyelination to coagulative necrosis (for review see Valk and Dillon, 1991). Among the described side-effects, radiation-induced optic neuropathy (Mihalcea and Arnold, 2008) and cognitive dysfunction (Greene-Schloesser and Robbins, 2012) are serious complications that can appear during the treatment or in a delayed manner. Recent study suggests that different white matter fibers have different sensitivity to radiation, which may contribute to the selective memory and learning dysfunction after brain irradiation (Nazem-Zadeh et al., 2012).

***Viral infection***. Progressive multifocal leukoencephalopathy (PML) occurs in immuno-deficient people and is caused by an ubiquitous neurotropic virus: the JC virus (for review, see Bellizzi et al., 2013). PML is mostly found in HIV patients and after grafts requiring immunosuppressive treatments. PML destroys OLGs, with intranuclear inclusions, leading to multifocal demyelination. The symptoms resemble those of multiple sclerosis (see below), including paralysis, vision loss, impaired speech, and cognitive deterioration, but the disease usually evolves much quicker.

#### Multiple sclerosis: a primary demyelinating disease

Genetic congenital hypomyelinating diseases have been detailed in a recent review (Perlman and Mar, 2012) and will not be treated in this review that focuses more specifically on acquired demyelinating diseases.

The main primary demyelinating disorder is multiple sclerosis (MS) which is a recurrent progressive disease. It is estimated that approximately 2.5 million people worldwide suffer from MS (Milo and Kahana, 2010). Most of them are young adults (aged 20–40 years old) with females outnumbering males by 2:1 ratio. MS is an auto-immune demyelinating and neurodegenerative disease of the CNS that imposes devastating neurological and psychiatric limits on patients. The specific symptoms are determined by the location of the demyelinated lesions. They may include loss of sensitivity, muscle weakness or spasms, ataxia, visual troubles, fatigue, incontinency, but also mood alterations (Compston and Coles, 2008). The aetiology of the disease is still not well understood. Both genetic and environmental factors play roles in the development of the disease (Ramagopalan et al., 2010). Overall, it has been estimated that changes in the human leukocyte antigen (HLA) system account for between 20 and 60% of the genetic predisposition (Baranzini, 2011). Environmental factors and particularly infectious agents that could be linked to MS have intensively been studied (for review, see Kakalacheva and Lunemann, 2011). Of all infectious agents, Epstein Barr virus (EBV) and human herpesvirus 6A have been most strongly associated with MS (Santiago et al., 2011; Almohmeed et al., 2013). Clinically, different forms of MS have been characterized (relapsing-remitting, primary or secondary progressive…), leading sometimes to the suggestion that the term MS could in fact bring together distinct pathologies, and several studies even challenge the dogma that auto-immunity is at the root of the disease suggesting that neurodegeneration could in fact come first (Trapp and Nave, 2008; Stys et al., 2012).

### Animal models of demyelination

Although *in vitro* models also represent useful tools to dissect mechanisms of myelination and to understand the interactions between the human immune system and the human CNS, we will focus here only on *in vivo* animal models (for review of *in vitro* models, see Jarjour et al., 2012; van der Star et al., 2012).

Animal models are rarely the exact counterpart of the human diseases, especially in pathologies as complex as multiple sclerosis, but they are very useful to get insights on the demyelinating/remyelinating processes. Indeed, no single model mimics all the features of the human disease but each may reflect specific aspects. Thus, the choice among the multiple demyelinating models should be determined by the research question.

Animal models of multiple sclerosis may be grossly divided in two groups: those that try to mimick with highest fidelity the human disease with its complexity, and those more reductionist that do not reproduce the whole pathogenesis of the disease but that are used for detailed dissection of specific mechanisms (Dubois-Dalcq et al., 2005). In the first category, we mainly find the experimental autoimmune encephalomyelitis (EAE) and viral models that are considered as clinically relevant models of MS. These are particularly interesting to study CNS inflammation, to perform pre-clinical safety studies for compounds targeting immune response and to analyze mechanisms of CNS viral infection (an encountered side effect of immunosuppressive treatments in MS patients). On the other hand, these models also present drawbacks: they induce small, disseminated, demyelination lesions with unpredictable locations (especially in the brain), which evolve in an asynchronous manner: demyelination and remyelination occur simultaneously, rendering difficult the analysis of temporal cellular and molecular changes. Toxin-induced demyelination models mainly represent the reductionist models. They present the advantage of generating reproducible extended demyelination lesions, with a clear temporal separation between de- and remyelination processes. They are thus useful to study specific roles of individual molecules in the repair process, and to develop strategies to promote remyelination. Since most of them are not inflammatory, they also allow the study of remyelination without the action of the adaptative immune system.

#### EAE and viral models

These models are clinically relevant models of MS characterized by mononuclear cell infiltration and demyelination (Traugott et al., 1986; Boyle and McGeer, 1990). They have been useful both in the demonstration of T cell-mediated demyelination and in the characterization of the pathogenesis of immune-mediated demyelinating disease.

***EAE***. In contrast to other models, EAE is more versatile and of purely autoimmune nature, but interestingly key pathological features of MS such as inflammation, demyelination, axonal loss and gliosis are also present in EAE.

EAE can be triggered by two approaches: either an active immunization using myelin peptides or a passive induction by adoptive transfer of activated myelin-specific Th1 or Th17 cells from immunized donors into naïve syngeneic recipients (for technical details see the review of McCarthy et al., 2012). Inflammation seems to be a more prominent feature than demyelination in EAE in which only small perivascular demyelination lesions are observed. The development of a modified “targeted EAE” model in which focal immune-mediated demyelination is triggered in a EAE rodent by stereotactic injection of TNFα in white matter tracts allowed to obtain larger localized and reproducible demyelination lesions (Kerschensteiner et al., 2004; Magalon et al., 2007). Another major drawback of the EAE model is the absence of B cell component in the disease process. Indeed, in MS patients B cells play an important role in the disease and autoantibodies are detected in blood, CSF and CNS lesions (Link et al., 1989). However, disease induced in B cell-deficient mice immunized is indistinguishable from that induced in wild-type mice (Hjelmstrom et al., 1998). By contrast, Wekerle created a transgenic mouse spontaneously developing a relapsing/remitting EAE but that requires the presence of B cells. These mice express specific T cell receptor for a MOG peptide and drive the production of auto-antibodies by endogenous B cells (Pöllinger et al., 2009).

EAE induction results in the development of an ascending paralysis. Clinical manifestations are due to a preferential attack on the spinal cord. The first signs appear 5 to 10 days after induction as a flaccid tail, then hind limb weakness is observed and finally paralysis of hind and front limbs. The disease may then develop into remitting/relapsing form or into a more chronic progressive form depending on the mouse strain and the induction protocol used. Mice that completely recover and remain stable after the first episode are referred as monophasic. Mice may also partially recover after the acute phase and keep a handicap (Batoulis et al., 2011; McCarthy et al., 2012).

EAE can also be used in species closer to humans such as non-human primates (t Hart et al., 2005). Marmosets and Rhesus macaque monkeys present the advantage of immunologic and physiology proximity but also of genetic diversity (outbred animals) and environmental living conditions (not specific pathogen free) more representative of the patient population. However, these aspects can also be considered as disadvantage since they introduce variability in the experiments and the number of primates used is generally quite low due to ethical and cost considerations. Nevertheless, these models remain a necessary step before leading a candidate drug toward clinical trial in human since several promising compounds identified in rodents showed no beneficial effects in MS patients.

***Viral models***. Considering the potential contribution of viral infections in the etio-pathogenesis of MS (see above), viral models may provide relevant information on the interactions between the immune and nervous systems in this pathology. However, these models are rarely used to dissect mechanisms of remyelination due to the complex interplay between immune and nervous systems. The most common viral models in rodents are Semliki Forest virus (SFV), Theiler's murine encephalomyelitis virus (TMEV) and mouse hepatitis virus (MHV). Virus may directly target neurons, thus triggering primary neuron loss and secondary demyelination (“inside out models” such as TMEV) or infect OLGs with primary demyelination and secondary axon loss (“outside in” models such as MHV) or act in an indirect manner provoking immune-mediated attack of the CNS (Atkins et al., 2000; van der Star et al., 2012). This is interesting since controversy still exist on the primary nature (neurodegeneration or demyelination) of the lesions in MS patients (Stys et al., 2012).

#### Toxin-induced models of demyelination

Stereotaxic injections of gliotoxic compounds such as lysophosphatidyl choline (also known as lysolecithin or LPC) and ethidium bromide are widely used to trigger focal demyelination. A comparative study of these different approaches showed that all compounds triggered little axonal damage (when the volumes injected remain small) but large demyelination lesions that underwent spontaneous remyelination with slightly different kinetics, remyelination being faster in rats treated with LPC compared to ethidium bromide (Woodruff and Franklin, 1999).

***LPC model***. LPC is a membrane-dissolving agent that mainly affects myelin a structure particularly rich in lipids. Most OLGs degenerate but OPCs are spared. Injection of 0.5–2 μl of 1% solution in white matter fiber tracts (often performed in spinal cord, corpus callosum or caudal cerebellar peduncle) triggers an ellipsoid demyelinated lesion over few mm^2^. Demyelination can be observed as soon as 3 days after LPC injection, reaches its maximum at day 7 and then the remyelination process takes over. The remyelination is complete after 14–21 days. Although this model is usually considered as non-inflammatory, it has been shown that cytokines such as MCP1, MIP1a, and TNFα are secreted after LPC injection and attract lymphocytes and macrophages that contribute to demyelination (Ousman and David, 2001). Injection of LPC in the corpus callosum proximal to the subventricular zone has been a very useful tool to study and demonstrate the recruitment of SVZ-derived progenitors to the demyelinated lesion and their contribution to remyelination (Nait-Oumesmar et al., 1999).

***Ethidium bromide***. Ethidium bromide is a DNA intercalating agent therefore it is not specific to OLGs: it also presents a cytotoxicity on astrocytes (Blakemore, 1982) and OPCs (Sim et al., 2002). Ethidium bromide chelates nucleic acids, causes oxidative stress (Abdel-Salam et al., 2012), and kills cells preserving axons. Initially used by intracisternal injections (Yajima and Suzuki, 1979), it is now essentially used as focal injections in spinal cord and cerebellar peduncle. Demyelination is delayed compared to LPC but lesions are bigger; remyelination is delayed as well, in part due to a deficit in debris removal in the ethidium bromide model (Shields et al., 1999). Irradiation (40 Gy) has been sometimes used in conjunction with ethidium bromide: it allows the depletion of endogenous OPCs over larger areas and to examine the migration potential of OPCs to repopulate the ethidium bromide-induced demyelinated lesion (Blakemore et al., 2002).

***Cuprizone model***. By contrast to LPC and ethidium bromide, Cuprizone is not a focal demyelinating model. Cuprizone is administered orally in the food and triggers widespread demyelination in white and gray matters although regional variability is observed (Skripuletz et al., 2008; Silvestroff et al., 2010; Schmidt et al., 2013; Steelman et al., 2013). The dose of 0.2% cuprizone is admitted to provide the best balance between morbidity and demyelination efficiency (Hiremath et al., 1998). This model is highly suitable for investigating the remyelination process without the influence of the peripheral immune system since there is no blood brain barrier damage, and it is robust, reproducible with well detectable de- and remyelination processes (for review see Skripuletz et al., 2011). Its main drawback is that the mechanisms of actions of cuprizone-induced demyelination are not well identified. Cuprizone [oxalic acid bis(cyclohexylidene hydrazide)] is a copper chelator, therefore the leading hypothesis is that cuprizone-induced demyelination is due to deficient copper concentrations (Benetti et al., 2010). Giant mitochondria are observed in OLGs after cuprizone treatment (Acs and Komoly, 2012). OLGs being particularly susceptible to altered energy metabolism, mitochondrial dysfunction could be largely responsible for cuprizone-induced demyelination. Several studies reinforced the interest in the cuprizone model by revealing a mitochondria involvment in both OLG apoptosis and tissue degeneration in MS (Kalman et al., 2007).

Complete demyelination is observed after 5 weeks of cuprizone feeding, however OLG death begins at the end of the first week already (Mason et al., 2004; Hesse et al., 2010). By contrast, there is an early increase in the number of microglia and astrocytes followed by OPCs proliferation, with a peak at 4.5 weeks (Matsushima and Morell, 2001). These OPCs start to differentiate into new OLGs even if cuprizone is still present (Gudi et al., 2009). When Cuprizone is stopped after 5 weeks, remyelination is completed within 2 weeks. However, if cuprizone is maintained on the long term (up to 12 or 16 weeks), the newly formed OLGs will be in turn destroyed (Lindner et al., 2009) and even OPCs will be affected (Mason et al., 2004). In this case, remyelination will be delayed until 12 weeks after cuprizone removal (Lindner et al., 2009).

Surprisingly, despite widespread demyelination, no overt neurological signs are detected in cuprizone fed mice. This is probably due to minimal axon damage in this model (Lindner et al., 2009). More sophisticated behavioral tests suggested motor coordination deficits (Liebetanz and Merkler, 2006; Franco-Pons et al., 2007; Skripuletz et al., 2010) and impairment in spatial working memory (Makinodan et al., 2009; Xu et al., 2009).

#### Irradiation

Although irradiation does not directly trigger demyelination, it kills OPCs (Hinks et al., 2001). Therefore, this approach has been extensively used in combination to focal demyelination in order to study remyelination after OPC depletion and to compare remyelination potential of endogenous vs. transplanted OPCs (Blakemore et al., 2000a). Fourty Gy irradiation is sufficient to inhibit endogenous myelin repair (Blakemore and Patterson, 1978), and transplanted OPCs engraft better and contribute more to myelin repair when endogenous OPCs have been totally depleted (Irvine and Blakemore, 2007).

#### Models of ischemia- and trauma-induced demyelination

As mentioned above, apart from primary demyelinating diseases, demyelination also occurs consecutively to trauma or white mater stroke. Clinical studies showed that atrophy of white matter tracts and subsequent demyelination is a common occurrence in patients with chronic trauma-induced demyelination. Furthermore, several studies have emphasized the progressive nature of lesions after white matter injury (Bramlett and Dietrich, 2007; Gouw et al., 2008). Most white matter infarcts are believed to be secondary to vascular occlusion from changes in small vessels, as well as endothelial cell dysfunction. Few animal models exist to reproduce traumatic axonal injury or white matter stroke (Sozmen et al., 2012).

***Traumatic axonal injury***. Fluid percussion injury in rats has become the most extensively used animal model to study human traumatic brain injury producing both focal and diffuse lesions. However, few studies have focussed on the demyelination aspect of the lesion. Following moderate fluid percussion on the parietal cortex, OLG number decreases as soon as 3 days after the lesion in the ipsilateral external capsule and corpus callosum (Lotocki et al., 2011b). Posttraumatic hypothermia can protect from OLG death by interfering with caspase3-mediated cell death mechanisms (Lotocki et al., 2011a), thus hypothermia may be a therapeutic strategy to protect from demyelination and minimize functional outcome of traumatic brain injury.

***White matter stroke***. Two main models are used to either mimick focal white matter strokes (consecutive to acute ischemia or occlusion of deep penetrating arteriole), or more generalized white matter disease (induced by dysregulated cerebral blood flow). This is respectively obtained by focal vasoconstriction (Hughes et al., 2003) or by artery ligature leading to massive ischemia (Ni et al., 1994; He et al., 1999). Selective damage of cerebral white matter has also been achieved using an hypoperfusion model in which common carotide arteries are narrowed by micro-coils (Shibata et al., 2004). Prolonged cerebral hypoperfusion triggers reproducible lesions with blood brain barrier disruption, oxidative stress, glial reactivity and oligodendrocytic loss. These models may be useful to study the time course of axonal injury and myelin disruption, and thus to establish critical therapeutic windows.

#### Genetic models of acquired demyelination

Several genetic mouse models have been developed to specifically kill OLGs in order to examine the consequences of primary OLG death without attack from immune system. The most frequently used systems are the expression of the active form of the diphteria toxine or its receptor under the control of OLG specific promotors (MOG, MBP, or PLP) (Buch et al., 2005; Pohl et al., 2011; Oluich et al., 2012). Altogether these genetic models show that induction of OLG death in adult mice is followed by clinical outcome 3–6 weeks postinduction characterized by spastic paralysis of the hindlimbs, tremor, ataxia, and kyphosis. At histological and cellular level, progressive myelin vacuolation, axonal damage and OPCs reactivity were consistently observed, with partial and heterogenous remyelination. However, although widespread demyelination is generally described, one study using a MBP- diphteria toxine receptor mouse together with diphteria toxine injection reported that despite loss of OLG cell bodies and alterations at the nodes of Ranvier, no overt demyelination was observed (Oluich et al., 2012). The authors thus suggest that axonal pathology occurring in the absence of demyelination can still be secondary to primary pathology of the OLG.

Interestingly, in all these studies despite the presence of myelin debris no anti-CNS immunity was initiated: no BBB permeability nor leukocyte infiltration was detected; by contrast, microglial activation was present (Pohl et al., 2011; Locatelli et al., 2012; Oluich et al., 2012). These studies indicate that OLG death *per se* does not trigger inflammation in the CNS.

The animal models described above, and more specifically toxin-induced demyelination models have largely contributed to our understanding of the demyelination and remyelination processes.

### Characterization of demyelination/remyelination processes

#### Spontaneous remyelination

Since the 70's multiple observations of MS patient's brain make up evidence that remyelination can follow demyelination (Prineas and Connell, 1979; Hirano, 1989; Patrikios et al., 2006). Remyelination can reach up to 90% in some patients whereas in others it is sparse and restricted to the marginal zone of the lesions; no correlation between the extent of myelin repair and the form or stage of the disease could be established (Patrikios et al., 2006). An extensive destruction of OLGs is observed in the acute MS, despite some preservation in some lesions. However, in the chronic MS a complete preservation of OLGs in the early stages and an extensive destruction and loss of OLGs in the late stages is observed (Ozawa et al., 1994). In later stage, when the OLGs in lesions suffered an extensive destruction, the remyelination is restricted to the lesional borders (Ozawa et al., 1994). The failure of the myelin repair is not only due to OLG loss, but also to the recurrent episodes of demyelination and inflammation in the same area. This repetition is considered as one of the most important causes of remyelination failure and OPC exhaustion (Prineas et al., 1993).

Overall, these studies show the existence of spontaneous remyelination in the human brain, but its efficiency depends on several factors and conditions such as the lesion location, demyelination extent, repetition and severity of attacks and genetic background of the patients.

***Morphological evidence of myelin regeneration***. Early groundbreaking electron microscopy studies revealed that regenerated myelin could be distinguished from normal myelin (Bunge et al., 1961; Gledhill and McDonald, 1977; Prineas and Connell, 1979; Blakemore and Murray, 1981; Hirano, 1989). The most obvious differences are the variations observed in myelin thickness and internode organization. Indeed, it has been reported that the g ratio (the fraction of the axonal circumference to the axonal plus myelin circumference) is increased indicating a reduction in myelin thickness, and the internode length is decreased during the regenerative process. During development, myelin thickness is positively correlated with axon diameter, whereas the thickness of myelin sheath generated during remyelination is reduced and independent of axon diameter (Fancy et al., 2011). Therefore, regenerated myelin is easy to recognize for large diameter axons but more difficult to analyze in small diameter axons such as in the corpus callosum (Stidworthy et al., 2003). Interestingly, a recent study challenged this dogma by showing that after a long delay (6 months), remyelinated axons are indistinguishable from unaffected axons, except for the largest ones (Powers et al., 2013). Whether the decrease in myelin thickness is due to a specific remyelination phenomenon or to an adult myelination property is an emerging and interesting question. A recent study revealed that in physiological conditions, the adult-born OLGs produce thinner and shorter myelin than myelin synthesized at earlier stage in neonate brain (Young et al., 2013). This observation suggests that the increase of the g-ratio in the remyelination is correlated with a specific property of the adult myelination.

***Contribution of parenchymal OPCs to spontaneous remyelination***. Following demyelination, most OLGs are eliminated and the few remaining ones do not appear to divide (Keirstead and Blakemore, 1997; Carroll et al., 1998), suggesting that they probably do not contribute to myelin repair. By contrast, many studies demonstrated the early reaction of OPCs following demyelination, showing proliferation and colonization of the lesion (Franklin et al., 1997; Levine and Reynolds, 1999; Blakemore et al., 2000b; Chari and Blakemore, 2002a). During remyelination, OLGs density increases at the expense of OPCs number, suggesting that reactive OPCs finally differentiate into myelinating OLGs. Genetic tracing finally provided the absolute proof of OPC contribution to myelin repair. Using PDGFRα-CreER^T2^ mice, Franklin's laboratory demonstrated that adult OPCs generate myelinating OLGs after toxin-induced demyelination (Zawadzka et al., 2010). Myelinating Schwann cells were also observed in CNS during the repair process (for review, see Zujovic et al., 2010). However, genetic lineage tracing revealed that most Schwann cells generated after demyelination were produced by OPCs (Zawadzka et al., 2010). Thus, in the adult central nervous system, OPCs represent the main source of cells for myelin repair.

Activation of parenchymal OPCs does not occur only after primary demyelinating lesions, but also consequently to any insult leading to secondary demyelination including trauma (Flygt et al., 2013) or consecutive to amyloid plaque deposition in Alzheimer's disease (Behrendt et al., 2013) (see above § Demyelination secondary to a primary non-demyelinating pathology). OPC activation requires their exit from quiescence, their proliferation and migration toward the lesion. The migration and repopulation by OPCs is slow, especially if large areas depleted in OPCs are present (for instance in the EB plus irradiation model, see above). In such case, interaction between OPCs and demyelinated axons is delayed and remyelination is less efficient, suggesting that the environment of the acutely demyelinating tissue provides the support required to achieve successful terminal differentiation (Blakemore et al., 2002). Indeed, many studies revealed that inflammatory cues play important role in OLG regeneration and remyelination (Arnett et al., 2001; Kotter et al., 2001; Bieber et al., 2003; Li et al., 2005). This led to propose the temporal mismatch hypothesis according to which non-remyelinated MS lesions exist because OPCs are destroyed during the destructive phase leading to wide areas devoided of OPCs. Since the repopulation of OPC depleted tissues is slow, OPCs reach demyelinated axons in a delayed time-window when inflammatory cues necessary to promotes OPC reactivation and differentiation have disappeared (Blakemore et al., 2002; Chari and Blakemore, 2002b).

***Contribution of SVZ-derived progenitors to spontaneous remyelination***. Recently, studies revealed that SVZ-derived progenitors also contribute to myelin repair. Menn and collaborators were the first to bring formal demonstration of the oligodendrogenic potential of SVZ stem cells (Menn et al., 2006). In physiological condition, a small subpopulation of type C cells in the SVZ express Olig2 and PDGFRα. These SVZ-derived OPCs migrate to the corpus callosum and form mature OLGs (Menn et al., 2006). After demyelination insult this potential is four-fold increased suggesting a role for SVZ-derived OPCs in myelin repair (Menn et al., 2006). The first indication of SVZ contribution to myelin repair was provided by the observation of ectopic migration of neuroblasts from SVZ to periventricular white matter in LPC and EAE models (Nait-Oumesmar et al., 1999; Picard-Riera et al., 2002). A recent study has shown that vascular remodeling after acute demyelination lesion in the corpus callosum favors the exit and migration of neuroblasts from their niche to the lesion site, blood vessels acting as migratory scaffold for ectopic migrating progenitors (Cayre et al., 2013). The oligodendrogenic potential of neuroblasts was corroborated by grafting experiments showing that purified SVZ neuroblasts transplanted in the corpus callosum of shiverer mice (mice lacking MBP expression) massively generate myelinating OLGs (Cayre et al., 2006). Finally genetic tracing studies using DCX-CreER^T2^ mice provided the proof of this endogenous lineage plasticity from neuronal to oligodendrocytic fate after demyelination insult and unveiled the role of Chordin in this process (Jablonska et al., 2010).

The human sub-ventricular zone of the lateral ventricles contains a ribbon of astrocytes, self-renewal and multipotential cells (Sanai et al., 2004). These stem cells and their progeny present similarities with those of rodents: they express the same markers such as glial fibrillary acidic protein (GFAP), nestin, the polysialylated form of the neural cell adhesion molecule (PSA-NCAM) and the epidermal growth factor receptor (Bernier et al., 2000; Weickert et al., 2000). In MS patients, SVZ reactivity (increased cell proliferation and density) has been observed associated with the presence of neuronal progenitors with migratory phenotype (Nait-Oumesmar et al., 2007). Furthermore, some of these neuronal progenitors (PSA-NCAM+) also expressed oligodendrocytic marker such as Olig2 and Sox10 (Nait-Oumesmar et al., 2007). These triple labeled cells were more frequent in periventricular lesions than in more remote lesions (Nait-Oumesmar et al., 2007), suggesting that SVZ contribution to myelin repair together with lineage plasticity observed in rodents may also be at play in humans. It is thus important to characterize the mechanisms regulating this process in order to develop new therapeutic strategies promoting remyelination. Interestingly, the therapeutic potential of SVZ-derived NSCs in MS is not restricted to cell replacement. These cells also play beneficial role through immunomodulation: they regulate inflammatory processes and thus present neuroprotective properties favoring regeneration (Pluchino et al., 2003, 2009).

#### Factors controling remyelination

Spontaneous remyelination can be influenced by physiological factors such as aging, exercice and enriched environment. Aging has been shown to inhibit the efficiency of the process, mainly delaying OPC recruitment and differentiation (Shields et al., 1999; Sim et al., 2002). The age-dependent decrease in spontaneous myelin repair in rodents has been attributed both to intrinsic properties of aging OPCs, for instance decrease in CREB signaling (Miyamoto et al., 2013), and impaired response of macrophages (Zhao et al., 2006) and monocytes (Ruckh et al., 2012) to demyelination, the presence of myelin debris inhibiting OPC differentiation (Kotter et al., 2006; Ruckh et al., 2012). To note, a large-scale analysis of postmortem MS patient brains paradoxically did not find any correlation between the age at disease onset and the extent of remyelination (Patrikios et al., 2006). Exercise and enriched environment have been shown to reduce functional impairment in EAE mice, to increase SVZ mitotic activity and SVZ-derived progenitor mobilization toward demyelinated lesions, and finally to promote the OLG fate of recruited progenitors (Magalon et al., 2007). Interestingly, moderate physical activity improves functional outcomes in MS patients (Petajan and White, 1999).

Identifying factors and signaling pathways involved in myelin repair and notably in OPC determination, proliferation, migration or maturation is essential for a deeper understanding of these processes and the development of new therapies. Several lines of evidence highlight the potential role of secreted and contact mediated factors on demyelination and remyelination processes (Table 1).

**Table 1:**
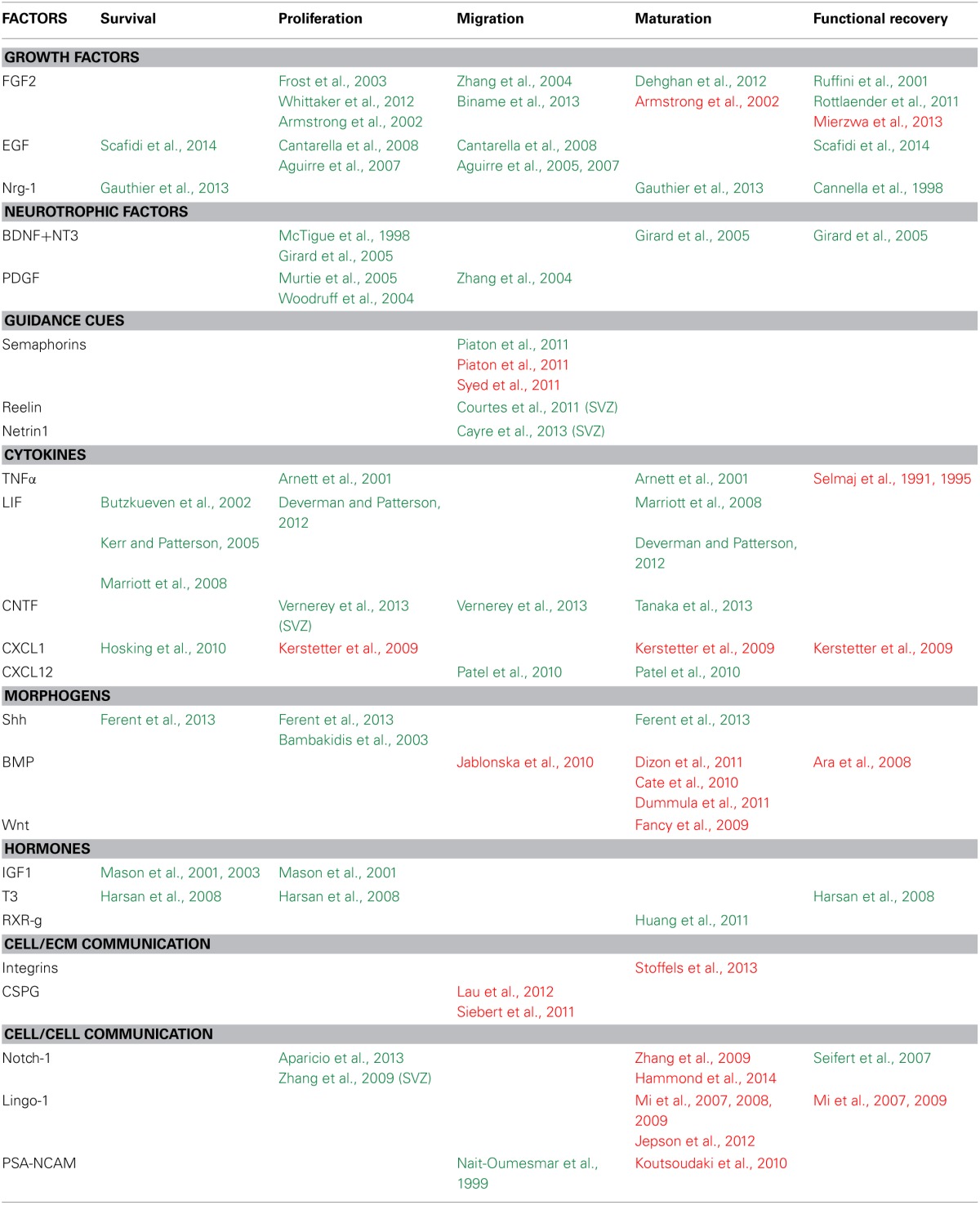
**Factors controlling the various steps of the regenerative process in mouse models of demyelination**.

References in green or in red indicate that the factor has respectively a positive or negative impact on the cellular processes or on functional recovery.

***Secreted factors***.

*Growth factors*. Fibroblast growth factor 2 (Fgf2) is expressed in the adult SVZ and plays a major role in the maintenance of NSCs (Zheng et al., 2004). Both Fgf2 and Fgf receptor 1 are up-regulated in the corpus callosum during cuprizone-induced demyelination and expressed by OLGs prior to remyelination (Zhou et al., 2012). Manipulations of Fgf expression or its signaling pathway affect multiple steps of the repair process. In viral (Frost et al., 2003), compressive (Whittaker et al., 2012) or cuprizone (Armstrong et al., 2002) induced demyelination, Fgf2 acts as a mitogen activating the proliferative response of OPCs. Fgf2 has been shown to trigger opposite effects on OLGs maturation depending on the model; while it favors OLG maturation following LPC induced demyelination of the optic chiasma (Dehghan et al., 2012) it blocks it in cuprizone and viral induced demyelination (Armstrong et al., 2002). The effect of Fgf on functional recovery also depends on the model, for example in EAE, Fgf reverts the clinical course and the pathological signs suggesting a neuroprotective effect (Ruffini et al., 2001; Rottlaender et al., 2011) while in cuprizone mouse model the presence of Fgf is associated with an increase in axonal damage (Tobin et al., 2011) and decrease in functional recovery (Mierzwa et al., 2013). *In vitro* analyses have also shown an effect of FGF on OPCs directed migration (Zhang et al., 2004; Biname et al., 2013) but no analysis have confirmed this result *in vivo* during demyelination.

Epidermal growth factor (Egf) can also induce proliferation of adult stem cells and neural progenitors in the adult brain (Doetsch et al., 2002). Over expression of this growth factor or its receptor confers new migratory properties to postnatal neural progenitors in the SVZ (Aguirre et al., 2005, 2007; Cantarella et al., 2008). In pathological condition, overexpression of human Egfr in OLG lineage cells or the administration of intranasal heparin-binding Egf immediately after injury has been shown to decrease oligodendroglia death, enhance generation of new OLGs from parenchymal OPCs and promote functional recovery (Scafidi et al., 2014). Intranasal heparin-binding Egf administration can also significantly increase SVZ cell proliferation and progenitor mobilization toward the lesions following LPC-mediated demyelination (Cantarella et al., 2008).

Neuregulin1 (Nrg-1) also signals through the EgfR ErbB. During development, Nrg-1-ErbB signaling is a key regulator of axon ensheathment and myelin thickness, regulating OLG differentiation, survival, maturation, and myelination (Kim et al., 2003; Brinkmann et al., 2008; Taveggia et al., 2008). In demyelination context, administration of Nrg-1 promotes functional recovery in acute and relapsing phases of EAE through the establishment of a more favorable immune environment (Cannella et al., 1998). In the same line, a significant increase in the number of newly formed OLGs was observed following SC injury upon Nrg-1 treatment (Gauthier et al., 2013).

Collectively, these data suggest that EgfR signaling promotes the various steps of the repair process and functional recovery.

*Neurotrophic factors*. NGF, BDNF, neurotrophin-3, 4, and 5 and their receptors influence myelin formation via two distinct mechanisms, either by acting on the neurons, changing the axonal signals that control myelination, or by acting directly on OLGs (Cellerino and Kohler, 1997; Wong et al., 2013). Several studies provide evidence that neurotrophins are effective positive regulators of remyelination and functional recovery after SC injury (McTigue et al., 1998; Girard et al., 2005). Transplantation of fibroblasts or Schwann cells expressing BDNF, GDNF, or NT-3 into demyelinated rodent SC enhances axonal growth, remyelination and locomotor recovery (McTigue et al., 1998; Blesch and Tuszynski, 2003; Girard et al., 2005). BDNF and NT3 in this context were shown to specifically stimulate OPC proliferation and differentiation (McTigue et al., 1998; Girard et al., 2005). PDGF-A has been shown to control OPC proliferation during the early phase of the regenerative process in LPC and cuprizone induced demyelination whitout favoring remyelination (Woodruff et al., 2004; Murtie et al., 2005).

*Guidance cues*. During development, the secreted class 3 semaphorins, Sema3A, and Sema3F, are known to have opposite effects on OPC migration: repulsive and attractive, respectively through distinct neuropilin co-receptors (NRP1 for Sema3A and NRP2 for Sema3F) (Sugimoto et al., 2001; Spassky et al., 2002). Sema3A and 3F and their receptors are up regulated in OPCs following LPC-induced demyelination. The lentiviral-mediated overexpression of Sema3F by astrocytes in and around the lesion increases OPC density within the lesion (Piaton et al., 2011) while the overexpression of Sema3A leads to an impaired OPC recruitment toward the demyelinated area thus preventing remyelination (Piaton et al., 2011; Syed et al., 2011). Reelin and Netrin1, two developmental guidance cues, are both upregulated after LPC induced demyeliation and promote SVZ-derived progenitor migration toward the lesion (Courtes et al., 2011; Cayre et al., 2013). Altogether, these results support a role of guidance cues in the repair process by regulating progenitor migration in the early phase of the regenerative process.

*Cytokines*. In CNS, cytokines exhibit critical role in repair processes by mediating inflammatory responses that promote pathogen clearance and prevent excessive tissue damage. However, their overproduction may lead to high inflammation and cell death (Mason et al., 2001). Interestingly, it is now clearly demonstrated that cytokines have a positive impact on the repair process.

Several studies have shown that the overexpression of TNFα worsens the demyelination in EAE mouse model (Selmaj et al., 1995) while the administration of anti-TNFα neutralizing antibodies has a protective effect (Selmaj et al., 1991; Selmaj and Raine, 1995). Despite this deleterious effect, some of these cytokines have been shown to have positive impact during remyelination phases by acting directly on OLGs (see Table 1). For example, administration of cuprizone to TNFα knockout mice leads to a significant delay in remyelination by decreasing the pool of proliferating OPCs and their maturation into OLGs (Arnett et al., 2001). This result clearly indicates a positive effect of TNFα during the repair process.

In the family of neurotrophic cytokines, ciliary neurotrophic factor (CNTF) and leukemia inhibitory factor (LIF) share some common protective roles in axons and oligodendrocytes. LIF exerts protective functions on oligodendrocytes and regulates positively many steps of the repair process (Butzkueven et al., 2002; Kerr and Patterson, 2005; Marriott et al., 2008; Deverman and Patterson, 2012). Beside its well-known neuroprotective action (Linker et al., 2009, 2002) CNTF has been shown to control directed cell migration during endogenous progenitor mobilization after demyelination (Vernerey et al., 2013). CNTF is strongly re-expressed around the lesion and acts as a chemoattractant on both SVZ derived progenitor and OPCs favoring their migration toward demyelinated areas (Vernerey et al., 2013). A distinct study has shown that decreased CNTF expression leads to OPC maturation defects after cuprizone-induced demyelination (Tanaka et al., 2013). Altogether, these results suggest that CNTF could positively regulate various steps of the regenerative process by promoting cell migration, proliferation and OLGs maturation.

CXCL1 and CXCR2 are detected in the SC in viral (Hosking et al., 2010) and autoimmune (Omari et al., 2009) demyelination models. Blocking CXCR2 results in an enhanced viral-induced demyelination associated with OLG death within the white matter tracts of the SC (Hosking et al., 2010). However, opposite results were obtained after chemical inhibition of CXCR2 either in EAE or LPC mouse model of demyelination (Kerstetter et al., 2009). In this context, the neutralization of CXCR2 significantly reduces lesion size and enhances remyelination suggesting that CXCR2 can also negatively influence remyelination in pathological context (Kerstetter et al., 2009). In cuprizone mouse model, CXCL12 (also named SDF-1) another chemokine secreted by astrocytes and microglia enhances both OPC mobilization and maturation within the corpus callosum (Patel et al., 2010). These findings support a dual role of cytokines in myelin repair following demyelination. It could be explained by the involvement of some cytokines and their receptors, such as CXCR2, in neuroinflammatory events acting on both hematopoietic and neural cells in inflammatory models of demyelination (Liu et al., 2010).

*Morphogens*. *Sonic hedgehog*. In the adult brain, Shh signaling is present (Traiffort et al., 1999; Coulombe et al., 2004) and required for the maintenance of stem cells niches (Charytoniuk et al., 2002) and migration of neuroblasts (Angot et al., 2008). The number of proliferating progenitors can be increased upon administration of Shh proteins in the cerebral cortex, corpus callosum (Loulier et al., 2005) and in the SC after injury (Bambakidis et al., 2003). Shh signaling pathway actors are expressed during the onset of demyelination in LPC induced demyelination (Ferent et al., 2013). In this context, Shh treatment increases the number of OPCs and mature myelinating OLGs at the lesion due to an enhanced proliferation, survival, and differentiation of the resident OPCs. Conversely, blocking Shh activity leads to a decrease of OPC proliferation and differentiation preventing repair (Ferent et al., 2013).

*Bone morphogenetic proteins*. Several studies have clearly demonstrated the implication of BMP signaling in healthy (Colak et al., 2008) and in demyelinated adult brain (Cate et al., 2010; Jablonska et al., 2010). Previous work in EAE model showed a correlation between the up-regulation of BMP signaling (BMP4, 6, and 7) in the SC and disease severity (Ara et al., 2008). The infusion of Noggin or chordin (BMP signaling inhibitors) increases SVZ cell commitment to OLG lineage and their mobilization in corpus callosum following LPC (Jablonska et al., 2010) or cuprizone (Cate et al., 2010) demyelination. Furthermore, BMP signaling decreases OLG maturation following perinatal hypoxic-ischemic brain injury (Dizon et al., 2011) or intraventricular hemorrhage (Dummula et al., 2011). Altogether these data indicate that BMP acts as a negative regulator of myelin repair by preventing cell mobilization and maturation.

*Wnt/β-catenin/Tcf4 signaling pathway*. During development, Wnt and BMP signaling pathways are known to have similar inhibitory effects on OLG differentiation (See and Grinspan, 2009). In this context, appearance of mature OLGs is delayed in mice in which Wnt/beta-catenin signaling is constitutively activated in OLGs. The transcription factor Tcf4, a mediator of Wnt signaling, has been shown to be expresssed within remyelinating lesions after LPC injection in the SC indicating activation of the Wnt pathway in the lesion (Fancy et al., 2009). Moreover, the overexpression of β-catenin in the OLGs induces a delay in OPCs differentiation and impaired remyelination in LPC model. Altogether, these experiments show that the Wnt/β-catenin/Tcf4 signaling pathway represents a negative regulator of remyelination.

*Hormones*. Various hormones have been shown to have beneficial effect on the repair process. Insulin growth factor-1 (IGF1) or triiodothyronine (T3)-treated mice exhibit enhanced OPC proliferation and survival after cuprizone intoxication (Mason et al., 2000, 2003; Harsan et al., 2008). T3 treatment leads to accelerated functional recovery (Harsan et al., 2008). The retinoid acid receptor RXR-γ is expressed during remyelination and its down regulation delays OLG differentiation and myelin repair (Huang et al., 2011). These examples suggest that treatments targeting hormones or their receptors could be valuable tools to promote remyelination.

In summary, secreted factor including growth factors, neurotrophins, morphogens, hormons and steroids (see Table 1) have been shown to have individual effects on remyelination by acting on one or several steps of the repair process. In the context of myelin repair, numerous studies have addressed the effect of combination of factors. For example a cocktail of factors including PDGFaa, bFGF, NT3, and IGF1 can increase OPC proliferation and migration in cuprizone induced demyelination (Kumar et al., 2007). EGF, bFGF and PDGFaa administration in SC after injury could promote the activation and oligodendroglial differentiation (Karimi-Abdolrezaee et al., 2012). Sequential administration of factors could represent a promising therapeutic strategy to regulate specific steps of the repair process. This is still an open field of investigation.

***Contact-mediated factors***.

*Cell/extracellular matrix (ECM) communication*. Ligands/ integrins complex are known to play a critical role in white matter development by promoting survival of newly formed OLGs that establish appropriate contacts with axons to be myelinated (Colognato et al., 2002). Almost all ligands for these αV Integrins including TenascinC and R, Fibronectin, and Vitronectin are transiently up regulated within demyelinated areas following toxin (LPC or EB)-induced demyelination (Zhao et al., 2009). Fibronectins are reported to inhibit OLG differentiation and remyelination in toxin-induced demyelination models (Stoffels et al., 2013). Interestingly, the overexpressed Fibronectin remains predominantly soluble following toxin-induced demyelination while aggregated forms of the Fibronectin are observed in EAE animals where remyelination often fails (Stoffels et al., 2013). These findings suggest that Fibronectin aggregation is mediated by inflammatory events and plays a critical role in the negative impact of this ECM.

Chondroitin and keratan sulphate proteoglycans represent the main inhibitory ECM molecules produced by reactive astrocytes in the glial scar following brain or SC injury (Fitch and Silver, 1997). An early and robust up-regulation of Chondroitin sulphate proteoglycan (CSPGs) was observed following LPC-induced SC demyelination (Lau et al., 2012). Moreover, the modulation of the CSPG synthesis or activity promotes the endogenous OPC recruitment toward the lesion site either in LPC-induced SC demyelination (Lau et al., 2012) or after SCI (Siebert and Osterhout, 2011; Siebert et al., 2011). These data suggest that, beside their positive effect in regenerative axonal sprouting, CSPGs located in the postinjury environment have a dual action, acting as a negative regulator of myelin repair by limiting OPC migration (Siebert et al., 2011).

*Cell/cell communication*. *Notch*. During development, the activation of Notch signaling was reported to have an inhibitory effect on OPC differentiation and myelination *in vitro* and *in vivo* (Wang et al., 1998; Givogri et al., 2002; Zhang et al., 2009). Notch1 is expressed by OPCs within lesions that undergo complete remyelination in SC of EAE animals (Seifert et al., 2007). Notch signaling is also detected in neural progenitors (Nestin+) and OPCs both in the SVZ and corpus callosum of LPC-demyelinated rats (Aparicio et al., 2013). Specific inactivation of Notch1 in OPCs in LPC-induced demyelination restricts OPC expansion and potentiates maturation and myelination (Zhang et al., 2009; Hammond et al., 2014). Nevertheless, another study got to a different conclusion following cuprizone-induced demyelination (Stidworthy et al., 2004). In this context, Notch1 depletion did not lead to any significant difference in remyelination parameters. Possible key difference in these findings could be the use of distincts Cre driver or the demyelination models used.

*LINGO-1*. LINGO-1 is a transmembrane signaling protein expressed by both neurons and OLGs. By using different strategies leading to the loss of LINGO-1 function, including Lingo1 gene knockout, infusion LINGO-1 antagonist (LINGO-1-Fc) and LINGO-1 si-RNA, Mi and collaborators have shown that LINGO-1 is an important negative regulator of myelination during development (Mi et al., 2005, 2008) and that its down regulation is important to control the timing of myelination onset during the repair process (Mi et al., 2007, 2009). A recent analyse has shown that LINGO-1 acts as both a ligand and a receptor and that the mechanism by which it negatively regulates OPC differentiation and myelination is mediated by a homophilic intercellular interaction. Disruption of this protein-protein interaction could lead to a decrease of LINGO-1 inhibition and an increase in myelination (Jepson et al., 2012). Consistent with these observations, an improved functional recovery was observed in LINGO-1 knock-out in the three main mouse models of demyelination (LPC, Cuprizone, and EAE) (Mi et al., 2009). Altogether, these data suggest a central role for LINGO-1 in orchestrating myelin repair acting as a negative regulator of OLG differentiation and myelination.

*PSA-NCAM.* During development, the polysialylated form of the neural cell adhesion molecule (PSA-NCAM) acts as an inhibitor of myelination, presumably by preventing myelin-forming cells from attaching to the axon (Doherty et al., 1990; Charles et al., 2000; Fewou et al., 2007). In the adult brain, PSA-NCAM expression is maintained in areas showing plasticity such as the SVZ (Durbec and Cremer, 2001; Rutishauser, 2008). Interestingly, previous studies have shown a transient re-expression of PSA-NCAM on myelinating precursor cells during their recruitment to demyelinated lesions in LPC-induced demyelination, which ceases when myelin repair was accomplished (Nait-Oumesmar et al., 1999). *In vitro* data have suggested that PSA-NCAM could be specifically involved in establishing the directionality of OPC migration in response to a concentration gradient of PDGF (Zhang et al., 2004). *In vivo*, the deletion of St8siaIV, one of the two polysialyltransferases enzymes responsible for the PSA synthesis, leads to an earlier remyelination in cuprizone mouse model (Koutsoudaki et al., 2010). These data suggest that PSA-NCAM could favor the mobilization of OPCs during early phase of the repair process but then would acts as an inhibitory signal for OPC differentiation.

#### From bench to bed

Despite recent progress in treating the inflammatory component of MS, current therapies have no clear impact on progression of disability related to myelin and axon injury. As described above, various factors can specifically regulate the cellular processes involved in remyelination including OPC migration, survival and proliferation and OLG maturation. Among these factors, those that have been shown to control OPC differentiation and could regulate the late phase of the repair process have received considerable attention and some are now promising candidate medicaments. For example, one approach currently in phase 1 clinical trial is the use of a neutralizing antibody targeting LINGO-1 since antagonism of LINGO-1 promotes OPC differentiation and leads to remyelination in mouse models of MS (Mi et al., 2007, 2009, 2013). Another promising candidate is olesoxime, a derivative of cholesterol shown to promote OLG maturation and myelin repair in animal models (Magalon et al., 2012). This compound is currently under phase 2 clinical trial for MS. The sphingosine-1-phosphate (S1P) receptor agonist, fingolimod (FTY720, Novartis) is in late stages of clinical development and may soon be approved to treat MS patients. This compound, beside its immunomodulating effect (Kataoka et al., 2005), has been shown to modulate S1P receptor activity promoting both OPC survival and OLG maturation (Jung et al., 2007; Miron et al., 2008). Altogether these compounds could represent future medicaments to favor myelin regeneration in human.

## Conclusion

Our knowledge of oligodendrogenesis has a great deal evolved over these past 10 years, with increasing number of research teams getting interested in postnatal production of OLGs and myelin repair. Interestingly, progenitor cells including parenchymal OPCs and SVZ cells represent more than 5% of the total cell number in the adult brain parenchyma. Such an important pool of progenitors may relate to high plasticity properties. Indeed, as mentioned in this review, oligodendrogenesis carries on throughout life, contributing to myelination and/or myelin remodeling, a process that may be important for functional plasticity, learning and memory. In parallel, many OPCs remain as quiescent or cycling very slowly progenitors, but with the ability to reactivate as soon as a demyelination insult occurs. Spontaneous myelin repair is therefore a strikingly common event, very efficient in most rodent models of demyelination, but quite variable in human patients suffering from autoimmune disease such as MS. The interplay between immune and nervous system is very complex in this pathology; inflammation acts like doctor Jekill and Mr. Hide: it is necessary to trigger OPC mobilization and differentiation, but deleterious for cell survival when it is prolonged. Because of this prevalent inflammatory component, existing treatments mainly exclusively target inflammation, with low efficiency on the progression of invalidity on the long-term. Neuroprotection, and thus remyelination needs to be promoted in conjunction to these treatments. A better knowledge of all the cell populations and factors involved in the repair process will undoubtedly allow the development of new therapeutic strategies.

### Conflict of interest statement

The Guest Associate Editor Antoine De Chevigny declares that, despite being affiliated to the same institution as authors El Waly, Macchi, Cayre and Durbec, the review process was handled objectively and no conflict of interest exists. The authors declare that the research was conducted in the absence of any commercial or financial relationships that could be construed as a potential conflict of interest.
